# Hyperandrogenemia in Early Adulthood Is an Independent Risk Factor for Abnormal Glucose Metabolism in Middle Age

**DOI:** 10.1210/clinem/dgab456

**Published:** 2021-06-21

**Authors:** Katri Tuorila, Meri-Maija Ollila, Marjo-Riitta Järvelin, Juha S Tapanainen, Stephen Franks, Katri Puukka, Terhi T Piltonen, Laure Morin-Papunen

**Affiliations:** 1 Department of Obstetrics and Gynecology, University of Oulu and Oulu University Hospital, Medical Research Center, PEDEGO Research Unit, Oulu, Finland; 2 MRC-PHE Centre for Environment and Health, Department of Epidemiology and Biostatistics, School of Public Health, Imperial College London, London, UK; 3 Center for Life Course Health Research, Faculty of Medicine, University of Oulu, Oulu, Finland; 4 Unit of Primary Health Care, Oulu University Hospital, OYS, Oulu, Finland; 5 Department of Life Sciences, College of Health and Life Sciences, Brunel University London, London, UK; 6 Department of Obstetrics and Gynecology, University of Helsinki and Helsinki University Hospital, Helsinki, Finland; 7 Institute of Reproductive and Developmental Biology, Imperial College London, London, UK; 8 NordLab Oulu, Department of Clinical Chemistry, University of Oulu and Oulu University Hospital, Medical Research Center Oulu, Oulu, Finland

**Keywords:** hyperandrogenemia, abnormal glucose metabolism, free androgen index, insulin resistance

## Abstract

**Context:**

The role of androgen excess as a contributing factor to abnormal glucose metabolism (AGM) and insulin resistance in women remains controversial.

**Objective:**

To investigate whether hyperandrogenemia (HA) estimated by serum testosterone (T) level and free androgen index (FAI) at ages 31 and 46 years is associated with insulin resistance, insulin secretion and AGM by age 46.

**Design:**

Prospective study including 5889 females followed at ages 31 and 46 years.

**Setting:**

General community.

**Participants:**

Women with HA were compared with normoandrogenic women at ages 31 and 46 years.

**Intervention:**

None.

**Main outcome measurements:**

AGM, including prediabetes and type 2 diabetes mellitus, homeostatic model assessments of insulin resistance (HOMA-IR) and of pancreatic β-cell function (HOMA-B).

**Results:**

At age 31 years, HA women displayed increased HOMA-IR (*P* = 0.002), HOMA-B (*P *= 0.007), and higher fasting insulin (*P *= 0.03) than normoandrogenic women after adjusting for body mass index (BMI). At age 46 years, there was a nonsignificant trend toward higher fasting glucose (*P *= 0.07) and glycated hemoglobin A1 (*P *= 0.07) levels in HA women. Women in the highest T quartile (odds ratio [OR] = 1.80; 95%CI, 1.15-2.82) at age 31 years and in the 2 highest FAI quartiles at ages 31 (Q4: OR = 3.76; 95% CI, 2.24-6.32) and 46 (Q4: OR = 2.79; 95% CI, 1.74-4.46) years had increased risk for AGM, independently of BMI, when compared with women in Q1. SHBG was inversely associated with AGM (at age 31 years: Q4: OR = 0.37; 95% CI, 0.23-0.60, at age 46 years: Q4: OR = 0.28; 95% CI, 0.17-0.44).

**Conclusion:**

Hyperandrogenemia and low SHBG in early and middle age associates with AGM independently of BMI.

Considering that type 2 diabetes mellitus (T2DM) is a major global health issue (1) predisposing to cardiovascular diseases, the most important cause of death worldwide, it is important to identify risk factors predisposing to abnormal glucose metabolism (AGM, including both prediabetes and T2DM). Among fertile aged women, HA is a common endocrine disorder (2, 3) that has been associated with an increased risk for T2DM, metabolic syndrome, and nonalcoholic fatty liver disease in some studies (4-7), although not all studies agree (8-11).

In women, androgen excess, hyperglycemia, and insulin resistance are intertwined through mechanisms that are not yet well understood. However, studies suggest this mechanism could be partially explained by the unfavorable effect of androgens on glucose uptake in women by promoting hepatic insulin resistance, reducing skeletal glucose uptake, and inducing oxidative stress (12-15). In addition, it is commonly recognized that insulin resistance induces compensatory hyperinsulinemia, which promotes ovarian androgen secretion (16). More specifically, insulin acts synergistically with LH, increasing testosterone (T) production from ovarian theca cells and inhibiting hepatic synthesis of SHBG, which leads to increased amounts of unbound, biologically active T (17).

Recent studies using rodent models have also suggested T excess causes prolonged activation of androgen receptor in pancreatic islet β cells, inducing insulin hypersecretion and eventually secondary β-cell failure, thus predisposing to T2DM (18-20). However, in the former literature, the association between androgens, insulin resistance, and T2DM in women is still not fully clarified (4, 9, 21-27). This may be due, at least partly, to the limitations of many published studies, such as cross-sectional design, unrepresentative clinic populations, different definitions of HA (use of serum levels of T vs free androgen index (FAI), use of cutoff values vs quartiles), and variable methods of steroid hormone measurement.

The aim of the present study was to investigate, for the first time to our knowledge, in a longitudinal population-based prospective data set, the association of HA in early adulthood (at age 31 years) and in middle age (at age 46 years), with AGM in middle age. In addition, we had access to a wide range of additional data allowing adjustment for several confounding factors.

## Materials and Methods

### Study population

**Figure 1.**
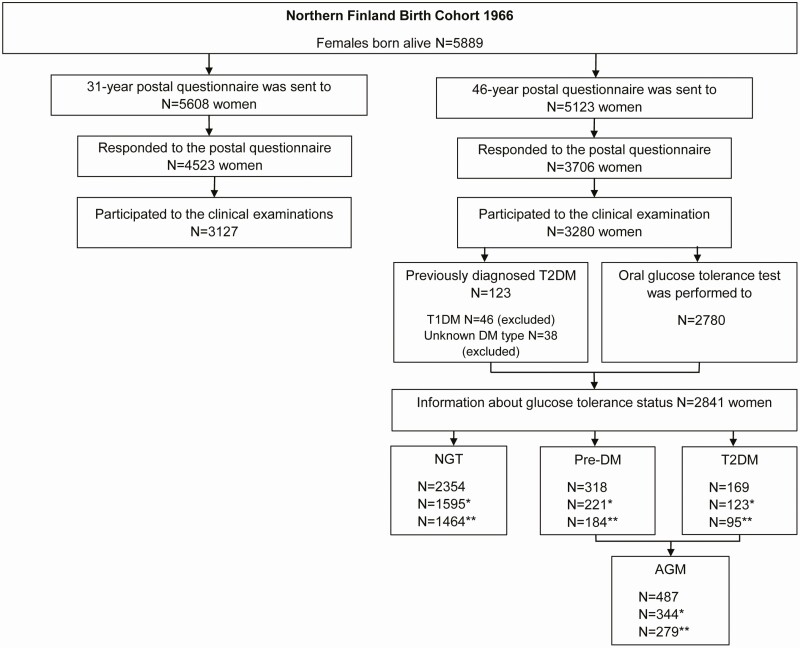
Flow chart of the study. Clinical examinations included for example blood sampling. *Women using hormonal contraceptive pills, having hormonal intrauterine device, or pregnant at age 31 years excluded. **Women using hormonal contraceptive pills, statins, hormonal replacement therapy, or having hormonal intrauterine device at age 46 years excluded. AGM, abnormal glucose metabolism; NGT, normal glucose tolerance; pre-DM, prediabetes; T1DM, type 1 diabetes; T2DM, type 2 diabetes.

The current study population arises from the Northern Finland Birth Cohort 1966, which is a large, longitudinal, prospective, population-based birth cohort comprising all individuals expected to be born in 1966, in the 2 northernmost provinces in Finland (Oulu and Lapland) (28). During that year, 5889 females were born alive, and thereafter the data have been collected at the ages 1, 14, 31, and 46 years (Fig. 1), also linking to multiple other data sources, including national registers. At age 31 years, a comprehensive postal questionnaire about reproductive health, work, and social background was sent to 5608 women, of whom 4523 (81%) responded. In addition, clinical examination, including anthropometric measurements and blood sampling, was performed in 3127 (76%) women. At age 46 years, another comprehensive postal questionnaire was carried out and 3706 (72%) of the contacted 5123 women responded. Clinical examination including measurements of height, weight, waist and hip circumference, together with blood sampling for hormonal and metabolic parameters, were performed in 3280 (64%) women. Body mass index (BMI) was calculated (kg/m^2^) by using measured height (average of 2 measurements) and weight (28).

### Laboratory methods

At ages 31 and 46 years, the serum levels of total T were measured using Agilent triple quadrupole 6410 liquid chromatography-mass spectometry (LC-MS) equipment with an electrospray ionization source operating in positive-ion mode (Agilent Technologies, Wilmington, DE). Multiple reaction monitoring was used to quantify T, using d3-testosterone, with the following transitions: m/z 289.2 to 97 and 289.2 to 109 for T and 292.2 to 97 and 292.2 to 109 for d3-testosterone.

At age 31 years, SHBG was assayed as previously described (29), and at age 46 years by chemiluminometric immunoassay (Immulite 2000, Siemens Healthcare, Llanberis, UK). As the SHBG analysis method changed over the course of the study, the SHBG values from age 31 years were transformed to be comparable with the SHBG values analyzed at age 46 years using this formula: 0.7615 × old method 31-year SHBG + 0.7088, and the results are reported according to this method (30).

At age 31 years, fasting plasma glucose were determined by glucose dehydrogenase method (Granutest 250, Diagnostica Merck, Darmstadt, Germany) and serum insulin levels by radioimmunoassay (Pharmacia Diagnostics, Uppsala, Sweden), and at age 46 years by an enzymatic dehydrogenase method (glucose) (Advia 1800, Siemens Healthcare Diagnostics Inc., Tarrytown, NY, USA) and by a chemiluminometric immunoassay (insulin) (Advia Centaur XP, Siemens Healthcare Diagnostics Inc.), respectively. To quantify the degree of insulin resistance, the homeostatic model assessment of insulin resistance (HOMA-IR) values were calculated using the validated calculator available at http://www.dtu.ox.ac.uk. In the regression analyses, we used HOMA-IR as a categorial variable with a cutoff value of 2.5 for normal vs abnormal (31, 32). To quantify pancreatic β-cell insulin secretion, the homeostatic model assessment of insulin of β-cell function (HOMA-B, continuous variable) was calculated using the equation 20 × insulin(µU/mL)/[Glucose (mmol/L) – 3.5]. Concentrations of glycated hemoglobin (HbA1c) and total hemoglobin were measured by an immunochemical assay method (Advia 1800; Siemens Healthcare Diagnostics Inc.) and their ratios were reported as mmol/mol. In the regression analyses, we used HbA1c as a categorial variable with a cutoff value of 48.0 mmol/mol for normal vs abnormal (33). FAI was calculated using the equation 100 × T (nmol/L)/SHBG (nmol/L). FSH concentrations were determined using an immunochemiluminometric method (Advia Centaur; both Siemens Healthcare Diagnostics).

### Definition of elevated T level

Elevated T level at ages 31 and 46 years was defined according to the normal upper limit for T at these respective ages based on the 97.5% percentile calculated in this population (2.3 nmol/L at age 31 and 1.7 nmol/L at age 46 years). This definition was based on the International Federation of Clinical Chemistry and Laboratory Medicine and Clinical and Laboratory Standards Institute guidelines regarding the formation of the reference values (34, 35).

### Definition of abnormal glucose metabolism

At age 46 years, a 2-hour oral glucose tolerance test (OGTT) was performed after an overnight (12-hour) fast in 2780 women. Plasma glucose levels were measured at the baseline and at 30, 60, and 120 minutes after the 75-g glucose load. Analyzed glucose levels were further classified according to World Health Organization standards into normal glucose tolerance (NGT), impaired fasting glucose, impaired glucose tolerance, and T2DM (36). Also, information about previously diagnosed T2DM was gathered from the postal questionnaire sent at age 46 years. All self-reported T2DM diagnoses were verified and completed from the hospital discharge registers and the national drug registers from the Social Insurance Institution of Finland. Presence of impaired fasting glucose, impaired glucose tolerance, or T2DM was classified as AGM.

### Statistical methods

Pregnant women and women using hormonal contraceptive pills or having a hormonal intrauterine device were excluded from the analyses at ages 31 and 46 years. In addition, women using hormonal replacement therapy (N = 101, 1.7%) or statins were excluded from the analyses at age 46 years.

Continuous data were presented as means ± SD or as medians for skewed distributions [25th percentile; 75th percentile]. The differences between continuous baseline parameters were analyzed using Student *t* test or Mann-Whitney *U* test, when appropriate. In the baseline analyses, the effect of BMI was estimated using general linear modeling. Categorial data were analyzed using cross-tabulation and Pearson’s chi-squared test.

We investigated the association between T and AGM, HOMA-IR, and HOMA-B using T as the categorial variable. SHBG and FAI were used as continuous variables. In addition, we divided T, SHBG, and FAI into quartiles at both ages 31 and 46 years and used the first (lowest) quartile as a reference group to study the associations with AGM and HOMA-IR at age 46 years.

Binary logistic regression models were used to estimate the factors associated with AGM and HOMA-IR and linear regression models to study factors associated with HOMA-B. The results of logistic models were reported as odd ratios (ORs) with 95% CIs and results of linear regression models as beta coefficients (Bs) with 95% CIs and *P* values. Model I included adjustment for BMI at age 46 and model II adjustments for BMI, smoking (never a smoker, former smoker for >6 months, former smoker for <6 months, and current smoker), education (basic, secondary, tertiary), and consumption of alcohol (classified as: not use, light use, moderate, heavy use) at age 46 years.

A *P* value < 0.05 was considered statistically significant. Statistical analyses were performed using IBM SPSS Statistics 22.0 (SPSS, Inc., 1989, 2013, IBM Corp.).

### Ethical approval

The study followed the principles of the Declaration of Helsinki. The Ethics Committee of the Northern Ostrobothnia Hospital District approved the study (decision number 94/2011 and 12/2003). All subjects have received written and oral information and gave their written consent to use all data.

## Results

### Baseline characteristics

#### Comparison of women with elevated and normal T levels at age 31 years

At age 31 years, women with elevated T levels (T > 2.3 nmol/L) tended to have higher BMIs (*P *= 0.07) and waist circumferences (*P *= 0.09) and were more often overweight or obese (BMI ≥ 25 kg/m^2^) than the women with normal T levels (48.8% [n = 21] vs 33.5% [n = 628], *P *= 0.04) (Table 1). Women with elevated T levels had also significantly higher levels of fasting insulin (*P *= 0.02), HOMA-IR (*P *= 0.03), and HOMA-B (*P *= 0.001) compared with women with normal T levels, independently of BMI (Table 1). Because the presence of T2DM is well-known to associate with impaired insulin secretion, we ran analyses after excluding the women with T2DM from the results concerning HOMA-B and HOMA-IR.

**Table 1.** Baseline characteristics in normoandrogenic and hyperandrogenic women at ages 31 and 46 years

At age 31 y				
	T ≤ 2.30 nmol/L (n = 1795-1877)	T > 2.30 nmol/L (n = 39-43)	Crude *P* value	BMI-adjusted *P* value
BMI (kg/m^2^)	23.21 [21.16; 26.24]	24.96 [21.17; 29.73]	0.07	
Waist (cm)	79.22 ± 12.11	85.03 ± 18.84	0.09	
BMI ≥ 25 (kg/m^2^), n (%)	628 (33.5)	21 (48.8)	0.04	
fP-Glucose (mmol/L)	4.93 ± 0.48	4.92 ± 0.51	0.95	0.47
fS-Insulin (mU/L)	7.93 ± 3.42	10.48 ± 7.54	0.02	0.03
HOMA-IR^*a*^	0.93 [0.76; 1.16]	1.00 [0.84; 1.40]	0.03	0.002
HOMA-B^*a*^	95.90 [83.30; 112.00]	106.50 [95.20; 124.00]	0.001	0.007
**At age 46 y**				
	**T ≤ 1.70 nmol/L** (n = 1676-1933)	**T > 1.70 nmol/L** (n = 42-53)	**Crude** ***P* value**	**BMI-adjusted** ***P* value**
BMI (kg/m^2^)	25.22 [22.73; 29.04]	25.35 [21.74; 28.80]	0.81	
Waist (cm)	86.95 ± 13.00	87.34 ± 16.15	0.83	
BMI ≥ 25 (kg/m^2^), n (%)	1015 (52.5)	27 (50.9)	0.82	
fP-Glucose (mmol/L)	5.37 ± 0.77	5.57 ±1.06	0.06	0.07
fS-Insulin (mU/L)	7.30 [5.10; 10.60]	7.00 [5.20; 10.70]	0.75	0.41
HOMA-IR^*a*^	1.60 [1.13; 2.27]	1.72 [1.17; 2.40]	0.71	0.56
HOMA-B^*a*^	81.58 [60.99; 111.47]	81.11 [58.33; 108.00]	0.84	0.91
HbA1c (mmol/mol)	36.18 ± 4.99	37.47 ± 7.83	0.37	0.07
2-h OGTT (mmol/L)				
0 h	5.35 ± 0.57	5.26 ± 0.39	0.30	0.45
2 h	5.77 ± 1.50	5.63 ± 1.65	0.56	0.77

The results are reported as mean ± SD or as median [25th percentile; 75th percentile]. The differences between the 2 study groups were analyzed by Student *t* test or Mann-Whitney *U* test when appropriate, and with cross-tabulation and *x*^*2*^ test. The effect of BMI was estimated using general linear modelling (analysis of covariance).

Abbreviations: BMI, body mass index; fP, fasting plasma; fS, fasting insulin; HOMA-IR, homeostatic model assessment of insulin resistance; HOMA-B, homeostatic model assessment of pancreatic β-cell function; HbA1c, glycated hemoglobin A1; OGTT, oral glucose tolerance test.

^*a*^We assessed HOMA-IR and HOMA-B after excluding women with diagnosis of type 2 diabetes.

Women with elevated T levels reported more often oligomenorrhea (51.9% vs 12.3%, *P* < 0.001), hirsutism (31.6% vs 12.8%, *P *= 0.02), and both symptoms (43.5% vs 4.6%, *P *< 0.001) than women with normal T levels.

#### Comparison of women with elevated and normal T levels at age 46 years

At age 46 years, women with elevated T levels (T > 1.7 nmol/L) did not significantly differ regarding their baseline anthropometric parameters or glucose metabolic indices from the women with normal T levels, although there was a nonsignificant trend toward higher fasting glucose (*P *= 0.07) and HbA1c (*P *= 0.07) levels in women with elevated T levels (Table 1). The prevalence of overweight/obesity did not significantly differ between women with elevated and normal T levels (50.9% [n = 27] vs 52.5% [n = 1015], *P *= 0.81). By age 46 years, 20.8% (n = 10) of the women with elevated T reported having experienced infertility problems compared with 18.6% (n = 368, *P *= 0.84) of the women with normal levels. Of note, by age 46 years, women with elevated and normal T had a comparable prevalence of menopause (estimated by an FSH level >30 IU (37, 38); 9.8% [n = 5] vs 13.2% [n = 278], *P *= 0.67).

The occurrence of type 1 diabetes mellitus (T1DM) and T2DM in the first-degree relatives was asked in the postal questionnaire at age 46 years. At that age, only 1.9% (n = 1) of the women with elevated T had a first-degree relative with T1DM compared with 3.9% (n = 84) of the women with normal T (*P *= 0.72). Similarly, 42.3% (n = 22) of the women with elevated T had a first-degree relative with T2DM compared with 31.5% (n = 680) of the women with normal T (*P *= 0.13).

### **Testosterone, SHBG, and FAI levels in women with NGT and AGM at ages 31 and 46 years** (Table 2)

Women with AGM at age 46 years had significantly higher levels of T at age 31 years (*P *= 0.03) but the difference vanished after adjusting for BMI (*P *= 0.15). Also, these women had significantly lower SHBG levels and higher FAI than the women with NGT both at ages 31 and 46 years, independently of BMI (at age 31: *P *< 0.001 for both; at age 46: *P *= 0.02 and *P *= 0.001, respectively).

**Table 2.** Levels of T, SHBG, and FAI at ages 31 and 46 years in women with NGT or AGM at age 46

At age 31 y				
	NGT at age 46 y (n = 994-1018)	AGM at age 46 y (n = 205-215)	Crude *P* value	BMI-adjusted *P* value
T (nmol/L)	1.05 ± 0.45	1.14 ± 0.54	0.03	0.15
SHBG (nmol/L)	51.45 ± 28.28	42.14 ± 39.56	<0.001	<0.001
FAI	2.39 ± 1.44	3.51 ± 2.18	<0.001	<0.001
**At age 46 y**				
	**NGT at age 46 y** (n = 1457)	**AGM at age 46 y** (n = 246)	**Crude** ***P* value**	**BMI-adjusted** ***P* value**
T (nmol/L)	0.90 ± 0.46	0.86 ± 0.34	0.24	0.50
SHBG (nmol/L)	63.99 ± 29.12	48.81 ± 34.88	<0.001	0.02
FAI	1.44 [1.05; 2.00]	1.93 [1.40; 2.78]	<0.001	0.001

The results are reported as mean ± SD or as median [25th percentile; 75th percentile]. The differences between the 2 study groups were analyzed by Student *t* test. The effect of BMI was estimated using general linear modelling (analysis of covariance).

Abbreviations: AGM, abnormal glucose metabolism, including prediabetes or type 2 diabetes; BMI, body mass index; FAI, free androgen index; NGT, normal glucose tolerance; T, testosterone.

### Association of elevated T with AGM, HOMA-IR, and HOMA-B

Elevated T at age 31 or at age 46 years was not associated with AGM at age 46 years (OR at age 31 = 2.09; 95% CI, 0.85-5.14; OR at age 46 = 0.60; 95% CI, 0.21-1.70), but at age 31 years, elevated T was significantly associated with increased HOMA-IR at age 46 years in the unadjusted model (OR = 2.68; 95% CI, 1.19-6.02).

Elevated T at age 31 years, but not at age 46 years, was positively associated with HOMA-B at age 46 years, also after adjusting for BMI (model I: B at age 31 = 0.21; 95% CI, 0.02-0.40, *P* < 0.03) and with higher HbA1c levels at age 46 years in unadjusted model (OR at age 31 = 5.35; 95% CI, 1.18-24.16).

### Association of SHBG with AGM, HOMA-IR, and HOMA-B

SHBG at age 31 years was inversely associated with AGM at age 46 years after adjusting for BMI (model I: OR = 0.99; 95% CI, 0.98-0.99) and at age 46 years, also after other adjustments (model II: OR = 0.98; 95% CI, 0.98-0.99). Both at ages 31 and 46 years, SHBG was inversely associated with HOMA-IR at age 46, also after adjustments (model II: at age 31, OR = 0.98; 95% CI, 0.98-0.99; model II: at age 46, OR = 0.98; 95% CI, 0.97-0.99).

At ages 31 and 46 years, SHBG associated inversely with HOMA-B at age 46 years after adjusting for BMI (model I: B at age 31 = -0.09; 95% CI, -0.15 to -0.04, *P *= 0.001; model I: B at age 46 = -0.12; 95% CI, -0.16 to -0.07, *P *< 0.001). Moreover, serum levels of SHBG at ages 31 and 46 years associated inversely with HbA1c levels at age 46 years in unadjusted models (at age 31, OR = 0.97; 95% CI, 0.94-0.99; at age 46 OR = 0.97; 95% CI, 0.96-0.99).

### Association of FAI with AGM, HOMA-IR, and HOMA-B

Free androgen index at ages 31 and 46 years associated positively with AGM at age 46 after adjustments (model II: at age 31, OR = 1.31; 95% CI, 1.19-1.43; model II: at age 46, OR = 1.07; 95% CI, 1.00-1.15). Similarly, FAI at ages 31 and 46 years was positively associated with HOMA-IR after adjustments (model II: at age 31, OR = 1.24; 95% CI, 1.14-1.35; model II: at age 46, OR = 1.18; 95% CI, 1.07-1.31).

Free androgen index at age 31 and 46 years was associated positively with HOMA-B at age 46 years also after adjusting for BMI (model I: B at age 31 = 0.06; 95% CI, 0.01-0.10, *P* = 0.010; model I: B at age 46 = 0.11; 95% CI, 0.02-0.08, *P *= 0.001). In addition, FAI at age 31 years associated significantly with HbA1c levels at age 46 years also after adjustments (model II: OR = 1.22; 95% CI, 1.01-1.47).

### Association of quartiles of T, SHBG, and FAI with AGM and HOMA-IR

We found an independent positive association between the highest T quartile at age 31 years and AGM at age 46 years. Furthermore, after adjustments, all 3 top quartiles of SHBG at ages 31 and 46 years associated inversely and third and fourth quartiles of FAI at ages 31 and 46 years positively with AGM at age 46 (Tables 3 and 4).

**Table 3.** Associations of T, SHBG, and FAI quartiles at age 31 y with AGM at age 46 y in women in Northern Finland Birth Cohort 1966

T				
	Quartile 1	Quartile 2	Quartile 3	Quartile 4
N	331	307	286	282
Crude	1.00	1.43 (0.95-2.17)	0.80 (0.50-1.27)	**1.95 (1.29-2.93)**
Model I	1.00	1.39 (0.90-2.14)	0.78 (0.48-1.26)	**1.83 (1.20-2.81)**
Model II	1.00	1.36 (0.87-2.13)	0.83 (0.51-1.37)	**1.80 (1.15-2.82)**
**SHBG**				
	Quartile 1	Quartile 2	Quartile 3	Quartile 4
N	321	300	285	278
Crude	1.00	**0.44 (0.30**-**0.64)**	**0.26 (0.17**-**0.40)**	**0.23 (0.15**-**0.37)**
Model I	1.00	**0.54 (0.36**-**0.79)**	**0.38 (0.25**-**0.60)**	**0.37 (0.23**-**0.60)**
Model II	1.00	**0.52 (0.35**-**0.78)**	**0.36 (0.22**-**0.57)**	**0.37 (0.23**-**0.60)**
**FAI**				
	Quartile 1	Quartile 2	Quartile 3	Quartile 4
N	306	310	315	302
Crude	1.00	1.49 (0.88-2.51)	**2.49 (1.52**-**4.08)**	**5.13 (3.21**-**8.19)**
Model I	1.00	1.40 (0.81-2.39)	**2.06 (1.23**-**3.43)**	**3.48 (2.13**-**5.69)**
Model II	1.00	1.47 (0.83-2.59)	**2.16 (1.27**-**3.70)**	**3.76 (2.24**-**6.32)**

Results are shown as ORs with 95% CIs in quartiles, when quartile 1 was used as the reference category. Model I: adjusted for BMI as a binary variable (cutoff at 25.0 kg/m^2^) at age 46 y. Model II: adjusted for smoking, education, consumption of alcohol, and BMI as a binary variable (cutoff at 25.0 kg/m^2^) at age 46 y. Boldfaced numbers indicate statistically significant.

Abbreviations: AGM, abnormal glucose metabolism, including prediabetes or type 2 diabetes; BMI, body mass index; FAI, free androgen index; T, testosterone.

**Table 4.** Associations of T, SHBG, and FAI Quartiles at age 46 y with AGM at age 46 y in women in Northern Finland Birth Cohort 1966

T				
	Quartile 1	Quartile 2	Quartile 3	Quartile 4
N	431	415	434	423
Crude	1.00	0.82 (0.56-1.18)	0.73 (0.50-1.06)	0.72 (0.49-1.05)
Model I	1.00	0.84 (0.57-1.23)	0.78 (0.53-1.14)	0.78 (0.53-1.15)
Model II	1.00	0.83 (0.56-1.24)	0.70 (0.47-1.06)	0.78 (0.52-1.17)
**SHBG**				
	Quartile 1	Quartile 2	Quartile 3	Quartile 4
N	429	430	428	416
Crude	1.00	**0.47 (0.33**-**0.65)**	**0.15 (0.10**-**0.24)**	**0.18 (0.12**-**0.28)**
Model I	1.00	**0.56 (0.40**-**0.79)**	**0.21 (0.13**-**0.33)**	**0.30 (0.19**-**0.47)**
Model II	1.00	**0.57 (0.40**-**0.82)**	**0.23 (0.14**-**0.37)**	**0.28 (0.17**-**0.44)**
**FAI**				
	Quartile 1	Quartile 2	Quartile 3	Quartile 4
N	421	421	428	433
Crude	1.00	1.50 (0.93-2.42)	**2.30 (1.47**-**3.61)**	**4.34 (2.83**-**6.65)**
Model I	1.00	1.29 (0.80-2.11)	**1.80 (1.14**-**2.88)**	**2.73 (1.75**-**4.25)**
Model II	1.00	1.39 (0.83-2.30)	**1.79 (1.10**-**2.90)**	**2.79 (1.74**-**4.46)**

Results are shown as ORs with 95% CIs in quartiles, when quartile 1 was used as the reference category. Model I: adjusted for BMI as a binary variable (cutoff at 25.0 kg/m^2^) at age 46 y. Model II: adjusted for smoking, education, consumption of alcohol, and BMI as a binary variable (cutoff at 25.0 kg/m^2^) at age 46 y. Boldfaced numbers indicate statistically significant.

Abbreviations: AGM, abnormal glucose metabolism, including prediabetes or type 2 diabetes; BMI, body mass index; FAI, free androgen index; T, testosterone.

The second quartile of T at age 31 years associated inversely with HOMA-IR at age 46 years after adjustments. In addition, all 3 top quartiles of SHBG both at ages 31 and 46 years associated inversely with HOMA-IR at age 46. Finally, at age 31 years, third and fourth quartiles of FAI and at age 46 years, all 3 top quartiles of FAI associated positively with HOMA-IR at age 46 (Tables 5 and 6).

**Table 5.** Associations of T, SHBG, and FAI quartiles at age 31 y with HOMA-IR at age 46 y in women in Northern Finland Birth Cohort 1966

T				
	Quartile 1	Quartile 2	Quartile 3	Quartile 4
N	360	345	334	303
Crude	1.00	**0.69 (0.49-0.97)**	0.97 (0.70-1.34)	1.03 (0.74-1.44)
Model I	1.00	**0.62 (0.43-0.90)**	0.86 (0.60-1.23)	0.95 (0.66-1.37)
Model II	1.00	**0.61 (0.42-0.90)**	0.84 (0.58-1.22)	0.94 (0.64-1.38)
**SHBG**				
	Quartile 1	Quartile 2	Quartile 3	Quartile 4
N	334	346	357	339
Crude	1.00	**0.45 (0.33-0.62)**	**0.24 (0.17-0.34)**	**0.17 (0.12-0.25)**
Model I	1.00	**0.57 (0.40-0.79)**	**0.39 (0.27-0.57)**	**0.30 (0.20-0.45)**
Model II	1.00	**0.59 (0.42-0.83)**	**0.39 (0.27-0.57)**	**0.30 (0.20-0.45)**
**FAI**				
	Quartile 1	Quartile 2	Quartile 3	Quartile 4
N	355	333	317	315
Crude	1.00	1.22 (0.82-1.81)	**2.52 (1.74-3.64)**	**4.18 (2.92-6.00)**
Model I	1.00	1.05 (0.69-1.60)	**1.81 (1.22-2.69)**	**2.35 (1.59-3.46)**
Model II	1.00	0.98 (0.64-1.52)	**1.78 (1.19-2.67)**	**2.27 (1.52-3.40)**

Results are shown as ORs with 95% CIs in quartiles, when quartile 1 was used as the reference category. Model I: adjusted for BMI as a binary variable (cutoff at 25.0 kg/m^2^) at age 46 y. Model II: adjusted for smoking, education, consumption of alcohol, and BMI as a binary variable (cutoff at 25.0 kg/m^2^) at age 46 y. Boldfaced numbers indicate statistically significant.

Abbreviations: AGM, abnormal glucose metabolism, including prediabetes or type 2 diabetes; FAI, free androgen index; HOMA-IR, homeostatic model assessment of insulin resistance; T, testosterone.

**Table 6.** Associations of T, FAI, and SHBG quartiles at age 46 y with HOMA-IR at age 46 y in women in Northern Finland Birth Cohort 1966

T				
	Quartile 1	Quartile 2	Quartile 3	Quartile 4
N	486	481	485	482
Crude	1.00	0.88 (0.66-1.16)	0.81 (0.61-1.08)	0.85 (0.64-1.14)
Model I	1.00	0.89 (0.65-1.21)	0.88 (0.65-1.20)	0.93 (0.68-1.27)
Model II	1.00	0.93 (0.67-1.29)	0.91 (0.66-1.27)	0.99 (0.72-1.38)
**SHBG**				
	Quartile 1	Quartile 2	Quartile 3	Quartile 4
N	488	480	483	484
Crude	1.00	**0.30 (0.23**-**0.39)**	**0.20 (0.15**-**0.27)**	**0.10 (0.07**-**0.15)**
Model I	1.00	**0.37 (0.28**-**0.50)**	**0.33 (0.24**-**0.45)**	**0.20 (0.14**-**0.29)**
Model II	1.00	**0.36 (0.27**-**0.49)**	**0.34 (0.24**-**0.47)**	**0.21 (0.14**-**0.30)**
**FAI**				
	Quartile 1	Quartile 2	Quartile 3	Quartile 4
N	479	488	483	484
Crude	1.00	**1.83 (1.27**-**2.63)**	**2.81 (1.98**-**3.98)**	**6.84 (4.89**-**9.56)**
Model I	1.00	**1.50 (1.03**-**2.20)**	**2.07 (1.43**-**2.99)**	**3.82 (2.67**-**5.45)**
Model II	1.00	**1.64 (1.10**-**2.45)**	**2.06 (1.40**-**3.05)**	**4.31 (2.96**-**6.27)**

Results are shown as ORs with 95% CIs in quartiles, when quartile 1 was used as the reference category. Model I: adjusted for BMI as a binary variable (cutoff at 25.0 kg/m^2^) at age 46 y. Model II: adjusted for smoking, education, consumption of alcohol, and BMI as a binary variable (cutoff at 25.0 kg/m^2^) at age 46 y. Boldfaced numbers indicate statistically significant.

Abbreviations: AGM, abnormal glucose metabolism, including prediabetes or type 2 diabetes; FAI, free androgen index; HOMA-IR, homeostatic model assessment of insulin resistance; T, testosterone.

### Persisting HA and the risk of AGM

The prevalence of AGM was not increased in women who were in the highest T quartile, either at ages 31or 46 years (n = 84), compared with those women who were in the lowest T quartile at both ages (n = 80) (OR = 1.13; 95% CI, 0.46-2.75). However, being in the highest FAI quartile at both ages (n = 110) was significantly associated with AGM (model II: OR = 5.83; 95% CI, 1.43-23.76) when compared with those who were in the lowest FAI quartile at both ages (n = 92). Moreover, women in the lowest SHBG quartile at both ages (n = 133) had increased risk for AGM (model II: OR = 4.97; 95% CI, 1.67-14.77) when compared with women in the highest SHBG quartile, at both ages (n = 129).

When performing the analyses, by substituting BMI with waist circumference (with a cutoff value of 80 cm), the results did not substantially change.

## Discussion

To our knowledge, this is the first and largest population-based, follow-up study investigating the association between hyperandrogenemia (expressed as elevated T levels and/or FAI) and glucose metabolism because we included not only T2DM, but also prediabetes, insulin resistance (HOMA-IR), insulin secretion (HOMA-B), and HbA1c levels. We were able to show significant associations between measured HA and SHBG at age 31 years with AGM at age 46 years. Further analysis revealed that the associations were not only driven by BMI, but also by hyperandrogenemia per se. Interestingly, elevated levels of T at age 31 years and FAI, at ages 31 and 46 years, were associated with increased insulin resistance and secretion, mostly independent of BMI.

The present results indicate that high T levels at age 31 years measured by LC-MS (LC-MS/MS), are associated with AGM in later life; compared with women in the lowest quartile at age 31 years, women in the highest T quartile at age 31 years had almost twice the risk for AGM at age 46 years. In line with this, our group has previously shown, in the same cohort, a positive association between elevated T and FAI levels at age 31 years with gestational diabetes (39). So far, previous studies have explored the relationship between sex steroid hormone levels and T2DM with conflicting results (4, 9, 21-27). Moreover, they have not considered prediabetic states. A former meta-analysis, including mainly cross-sectional studies, reported that high levels of T were associated with an increased risk of T2DM in women (40). Similarly, a recent, large, retrospective study reported that the risk of T2DM started to increase significantly when serum T exceeded 1.5 nmol/L, with the highest risk in women with serum T ≥3.5 nmol/L (4). However, later studies with follow-up periods of 5 to 11 years, using LC-MS/MS, could not confirm this finding (9, 27) and a previous systematic meta-analysis, including 13 prospective studies, did not show any association between total T and T2DM (9). Of note, most of the studies included in the meta-analysis did not use LC-MS/MS. Although there is still a debate (41) about the most appropriate method of measurement, the current consensus is that T measurement in women should be performed using LC-MS/MS (42). This issue might explain the discrepancy between our results and those of the aforementioned meta-analysis. Another important difference is that the present study included a wider spectrum of glucose metabolism abnormalities because it also included prediabetes.

Our study showed a positive association of FAI with HOMA-IR, HbA1c levels and AGM, even after BMI adjustment, and an even tighter association for the women with persistently raised androgen levels (ie, being in the highest FAI quartile both at ages 31 and 46 years). In previous studies, the link between FAI and T2DM has been controversial. O’Reilly et al. reported a strong positive relation (4), and Muka et al. reported a positive association that vanished after adjustments for age, fasting status, insulin, glucose, and BMI (9). Even though FAI is not fully trouble-free in the evaluation of HA in women, it is commonly used and considered as a reliable proxy for bioavailable T (43, 44). All in all, these findings suggest that HA, at least as evaluated by FAI, is associated with AGM. Moreover, the present results strengthen the case for a pivotal effect of weight as a risk factor as well as the existence of a complex synergistic interrelationship between HA, obesity, and the risk of AGM through life.

In the present study and in line with previous literature (45, 46), SHBG was associated inversely with insulin resistance, insulin secretion, and AGM, independently of BMI. The synthesis of SHBG is influenced by several hormonal and metabolic factors, including insulin, which inhibits hepatic secretion of SHBG (47, 48). In line with our results, a prospective 6-year follow-up analysis from 3 Finnish population-based cohorts (including the Northern Finland Birth Cohort 1966) indicates that circulating SHBG is predictive of the degree of insulin resistance and glycemia and that elevated SHBG has protective role on T2DM risk (49). These findings suggest that SHBG may not be only a passive carrier protein but may also have an active function and play an independent role in the pathogenesis of T2DM. Further studies are needed to investigate whether the level of SHBG could be used as a predictive factor to identify subjects at risk for T2DM.

Interestingly, at age 31 years, the women with elevated T displayed significantly increased insulin resistance and secretion (expressed as greater HOMA-B, HOMA-IR, and insulin values) compared with the normoandrogenic women, independently of BMI. Also, at age 31 years, the glucose levels remained in the normal range and did not differ from those in normoandrogenic women, suggesting that the increase in insulin secretion was able to compensate worsened insulin resistance. Further, the 2 highest FAI quartiles at age 31 years associated with increased insulin resistance (HOMA-IR) and risk of AGM at age 46 years. Last, compared with the women with NGT, the women with AGM at age 46 years were more hyperandrogenic at ages 31 and 46 years because they had higher levels of T at age 31 and higher FAI both at age 31 and 46. This finding fits well with the results of previous studies about the possible mechanism linking HA and abnormal glucose metabolism. In mice, T excess has been shown to cause a chronic androgen receptor activation in pancreatic β cells, producing insulin hypersecretion and eventually secondary β-cell failure, which may predispose to T2DM (18, 19). In line with this hypothesis, at age 46 years, the women with excess androgen seemed to have limited capacity to increase insulin secretion as their HOMA-B levels were comparable to those of normoandrogenic women at age 46 and their fasting glucose and HbA1c levels tended to be higher than those found in normoandrogenic women. This could be seen as the onset of impaired β-cell insulin secretion. However, we did not detect significant difference in the 0-hour and 2-hour glucose levels during the 2-hour OGTT between normoandrogenic and hyperandrogenic women at age 46 years, which might be due to the relatively small number of the subjects in the analysis.

Another interesting observation was that the correlation between HA and glucose metabolism disorders observed at age 31 years disappeared at age 46 years. One explanation might be that the higher prevalence of overweight/obesity observed in women with HA at age 31 years was not present any longer at age 46 years. An additional explanation could also be the decline in androgen levels with time, decreasing also its adverse effect on glucose metabolism (50). Last, there might be also some unknown explanatory factors that could not be clarified in the present study setting. All in all, although the design of our study does not allow us to draw conclusions about causality or mechanism, these results are of interest regarding an early identification of women at risk for glucose metabolism disorders.

This study enhances our knowledge of the involvement of HA in disordered glucose metabolism by providing a large unique prospective dataset with unselected, homogenous study population. Other strengths of this study are the long follow-up period as well as high participation and response rates and low dropout rate. Furthermore, anthropometric parameters were, mostly, directly clinically measured. In addition, T measurements were performed using LC-MS/MS, the gold standard method (42). The OGTT was performed in a large subgroup of the study population at age 46 years, allowing the diagnosis of both prediabetes and previously undiagnosed T2DM. The inclusion of both prediabetes and T2DM as main outcomes is clinically important, as between 70% and 90% of prediabetic people will develop T2DM (51, 52). We were also able to analyze the effect of many confounding factors, such as the occurrence of family history of T1DM and T2DM, which did not differ between women with normal and elevated T levels at age 46 years. Last, our results allow us to consider possible mechanisms driving disordered glucose metabolism in women.

The limitations of this study include the use of only serum T as a marker of HA, even though other androgens, such as adrenal androgens, have a place in the evaluation of androgenicity in women. Over the course of the cohort’s follow-up, the laboratory method used in the evaluation of SHBG changed, and we had to use a conversion formula to make the FAI results from age 31 and 46 years comparable. However, the current method using conversion formula calculated by linear regression analysis was the least biased way to make the FAI results at ages 31 and 46 years comparable. Also, a recent use of contraceptive pills might have affected the SHBG level because we do not have information about the time when the women came off the contraceptive pill. As some of the CIs in the analyses were rather wide, some analyses may have been underpowered. Further, larger studies are therefore needed to confirm our results.

## Conclusion

The present study showed a positive association between early adulthood HA and abnormal glucose metabolism in middle age. In addition, there was a significant inverse association between SHBG and AGM, independently of BMI, suggesting that levels of SHBG could help identifying women at risk of disordered glucose metabolism. Our results also underline the complexity of the interrelationship of androgen excess with weight, insulin resistance, and insulin secretion. The present results also emphasize the importance of weight management early on, particularly in hyperandrogenic women, to reduce the risk for glucose metabolism disorders. Young women with HA and overweight or obesity should also be assessed regularly for glucose tolerance; however, whether HA remains a metabolic risk later in adulthood remains elusive. In the future, new studies should be designed to estimate whether therapeutic reduction of T excess in early adolescence is beneficial in decreasing the risk of glucose metabolism disorders later in life.

## Data Availability

Some or all datasets generated during and/or analyzed during the current study are not publicly available but are available from the corresponding author on reasonable request. The cohort center grants the final study permit.

## Abbreviations

AGM: abnormal glucose metabolism
BMI: body mass index
FAI: free androgen index
HA: hyperandrogenemia
HbA1c: glycated hemoglobin
HOMA-B: homeostatic model assessments of β-cell function
HOMA-IR: homeostatic model assessments of insulin resistance
LC-MS: liquid chromatography-mass spectometry
LC-MS/MS: tandem liquid chromatography-mass spectometry
NGT: normal glucose tolerance
OGTT: oral glucose tolerance test
OR: odds ratio
T: testosterone
T1DM: type 1 diabetes mellitus
T2DM: type 2 diabetes mellitus.

## Additional Information

***Disclosures:*** None of the authors have any conflict of interest.
